# Evaluation of Lipid Quality in Fruit: Utilizing Lipidomic Approaches for Assessing the Impact of Biotic Stress on Pecans (*Carya illinoinensis*)

**DOI:** 10.3390/foods13070974

**Published:** 2024-03-22

**Authors:** Lingyuan Zhou, Wei Zhang, Qingyang Li, Maokai Cui, Danyu Shen, Jinping Shu, Runhong Mo, Yihua Liu

**Affiliations:** 1 Research Institute of Subtropical Forestry, Chinese Academy of Forestry, Fuyang 311400, China; y2213879621@163.com (L.Z.); zwlzhi@126.com (W.Z.); 12316044@zju.edu.cn (Q.L.); m18909189109@163.com (M.C.); shen@caf.ac.cn (D.S.); shu_jinping001@163.com (J.S.); 2 Quality Testing Center for Non-Wood Forest Products of National Forestry and Grassland Administration, Chinese Academy of Forestry, Fuyang 311400, China; 3 Institute of Pesticide and Environmental Toxicology, Key Laboratory of Biology of Crop Pathogens and Insects of Zhejiang Province, Ministry of Agriculture Key Laboratory of Molecular Biology of Crop Pathogens and Insects, Zhejiang University, Hangzhou 310058, China

**Keywords:** pecan, lipidomic, lipid profile, insect damage, biotic stress, nutrition

## Abstract

There is a scarcity of data on how the lipid composition of oily seeds changes in response to biotic stress. Yellow peach moth (*Conogethes punctiferalis*) has caused massive economic losses on the pecan (*Carya illinoinensis*) industry. Lipidomics is used in this study to determine the lipid composition of pecan and how it changes in response to insect attack. Pecan had 167 lipids, including 34 glycerolipids (GL), 62 glycerophospholipids (GP), 17 fatty acyls (FA), 41 sphingolipids (SP), and 13 saccharolipids (SL). The effects of biotic stress on lipids, particularly GL and GP, were significant. Biotic stress significantly reduced the lipid content of chains longer than 48. Forty-four significantly different lipids were discovered as potential biomarkers for distinguishing non-infected pecans from infested pecans. In addition, we used bioinformatics to identify the five most important metabolic pathways in order to investigate the processes underlying the changes. Our discoveries may offer valuable insights for enhancing pecan production in the future and contribute novel perspectives towards enhancing the nutritional value of pecans.

## 1. Introduction

The pecan [*Carya illinoinensis* (Wangenh.) K. Koch] nut is a species in the Juglandacea family, and it is one of the oldest and most important nuts worldwide, second only to the almond and walnut [1]. Pecan is a prominent tree nut known for its flavor and nutritional value. Lipids play a key role in determining their overall quality and health benefits. Due to its abundant unsaturated fatty acids and beneficial bioactive phytochemicals, incorporating pecans into your diet has been associated with numerous health benefits such as improved cardiovascular well-being, lowered cholesterol levels, and a decreased likelihood of developing heart-related conditions [2,3]. Multiple research studies indicate that the quality of pecans can be negatively affected by various factors occurring before and after harvesting, leading to a decrease in their overall lifespan [4]. Growing variables, including climate, soil composition, and agricultural practices, can have a major impact on the fatty acid (FA) composition of pecans [5,6,7]. Genotypic variations among pecan cultivars also influence the FAs profile [8]. Furthermore, it is believed that high amounts of unsaturated fatty acids (UFA) have a substantial impact on pecan quality features during storage [9]. As a result, many scholars have undertaken extensive research on the changes in FAs and other quality components in pecan storage, as well as the relevant control measures [10]. However, past research on lipid alterations or deterioration in pecan kernels has mainly focused on total lipids, fatty acids, or volatiles derived from fatty acids [10], leaving little information on other lipid compositions in pecan.

As a significant ingredient of membranes, lipids comprise a vast array of complex compounds (divided into eight types) that perform critical functions in a variety of biological processes. Other lipids, such as phospholipids, glycolipids, and sterol lipids, have been shown to have strong biological activity in clinical and biological studies [11,12]. Lipidomics employs mass spectrometry as a sophisticated technique to unveil insights into the arrangement of complete lipid molecules within biological substances. Lipidomics has recently been used to assess the lipid content of many oily nuts [10,13,14]. This method may also be used to study the lipid changes that occur in pecans during storage [1,10]. However, there has been minimal success in characterizing the lipid composition in edible vegetable oil utilizing lipidomic analysis [15,16].

Pecan trees are susceptible to a variety of insect pests that can cause significant harm to both the foliage and the nuts. One of the most devastating pecan pests is the Yellow peach moth (*Conogethes punctiferalis*). Insect infestations can have a major impact on the quality and productivity of pecan crops, resulting in financial losses for growers [17]. Because of the possible consequences for nutritional quality, flavor, shelf life, and overall pest resistance, the influence of insect stress on the lipid metabolism and composition of oilseeds has received increased attention [16]. It has been shown that biotic pressures such as insect pests affect the fatty acid composition and several other quality components of oilseeds. Lipidomics offers useful tools for studying the complex lipid changes caused by insect stress in oilseeds. Data on changes in the lipid composition (based on lipidomics) of oilseeds in response to biotic stresses, on the other hand, are scarce [16]. To fill this knowledge gap, we identified the lipid species in non-infested and infested pecans in the current study. The purpose of this study was to (1) compare variations in the lipid composition of pecan seeds infested with or free of yellow peach moths; (2) assess the impact of biotic stress on pecan; and (3) delineate alterations in the lipid composition and associated metabolic pathways of three types of pecans. The current study’s findings will be useful in understanding the influence of biotic stress on changes in the lipid profile of pecans and improving pecan nutritional quality.

## 2. Materials and Methods

### 2.1. Chemicals and Reagents

Acetonitrile, methanol, formic acid, ammonium formate, and isopropanol (LC-MS grade) were purchased from Fisher Scientific (Hampton, NH, USA). Dichloromethane and isopropanol (HPLC grade) were obtained from Sinopharm Chemical Reagent Limited Corporation. Internal standards (details can be seen in Table S1) were purchased from C/D/N Isotopes Inc. (Pointe-Claire, QC, Canada) and Avanti Polar Lipids (Alabaster, AL, USA).

### 2.2. Preparation of Pecan Samples

The material for this study was obtained from the *Carya illinoinensis* Experimental Base in Dongtai City, Yancheng City, Jiangsu Province, China, and the variety was ‘Mahan’. During the commercial mature stage, all samples (about 20 kg) were collected in 2022 from 10- to 12-year-old trees in the Base. At each collection site, 20–25 pecans were taken from the top and lower sections of each tree, as well as from all four directions (east, south, west, and north). In some of the collected samples, insects were observed within the holes (Figure S1), and it was identified as second instar of *Conogethes punctiferalis* [18]. Based on visual inspection of insect holes in the pecan branch shells and fruit shells, pecans were classified into three types (Type A, B, and C, as shown in Figure S1). Type A represented non-infested pecans from non-infested branches, Type B represented non-infested pecans from infested branches, and Type C represented infested pecans from infested branches. To obtain representative samples, the pecan samples taken for every type were separated and properly mixed. The biological replicates for the three types of samples were 3 (A), 3 (B), and 6 (C). The pecan shell was manually broken after the green peel was mechanically removed. Finally, the pecan kernels were kept at −80 °C until analysis.

### 2.3. Lipid Extraction Procedure

A 5 g sample of crushed pecan was weighed and extracted with petroleum ether (b. p. 30–60 °C) in a Soxhlet Extractor (Soxtec™ 8000, Foss Analytical, Hillerod, Denmark). The oil sample (100 μL) was accurately weighed, and the MTBE (methyl tert-butyl ether)/methanol (4:1, *v*/*v*) was added and carefully mixed. A solvent combination of MTBE and methanol was employed to extract lipid components from the oil, with the goal of generating a two-phase solution system following extraction [19]. The mixture is then allowed to stand for 10 min at 4 °C. The residue was reconstituted in 200 μL of dichloromethane/methanol (1:1, *v*/*v*) (containing an internal standard) before being analyzed by UHPLC-HRMS/MS.

### 2.4. Instruments and Methods

Lipids were separated by a Waters CSH C18 column (2.1 mm × 100 mm, 1.7 μm) using a Thermo Fisher Ultimate 3000 UHPLC system (Thermo Fisher Scientific, Rockford, IL, USA). A ThermoFisher Q Exactive Hybrid Quadrupole Orbitrap Mass Spectrometry Mass Spectrometer was selected for the analysis of the eluted lipids using heated electrospray ionization in both positive (HESI+) and negative (HESI−) ionization modes. The mobile phases of the positive ionization mode were ultra-pure water (A) and isopropanol/acetonitrile (9:1, *v*/*v*) (B), each containing 10 mM ammonium formate and 0.1% formic acid, whereas the identical mobile phases, except for formic acid, were used in the negative ionization mode. Table S2 used a linear elution technique to separate lipids. Heated electrospray ionization was used to evaluate the eluted lipids in both positive and negative ionization modes. The Supplemental Materials (Table S2) include detailed information on instrument parameters and programming settings.

### 2.5. Lipid Identification

The peak detection, alignment, and compound identification of raw data were processed using Compound Discoverer (version 3.3, Thermo Fisher, Thermo Fisher Scientific, Rockford, IL, USA). In addition to the default parameters, the mass tolerance was set to 5 ppm and the retention time tolerance was 0.2 min. Compound identification was performed by searching the accurate *m*/*z* of MS1 and MS/MS spectra against LipidBlast database (VS68) and mzCloud database with matching threshold larger than 60%. Differential compounds were manually reviewed or re-identified. The finished data were exported in the form of a Peak Table file, containing mainly the observations (sample names), the variables (rt_mz), and the peak regions. Prior to conducting univariate and multivariate statistics, the data were normalized against the internal standards.

### 2.6. Quality Assurance

In order to create a quality control sample (QC sample), the pecan samples were combined in equal proportions. Throughout the analysis process, one QC sample was inserted for each of the 10 samples to be analyzed for repeatability. The high consistency observed in the total ion flow, including retention time and peak strength, of the QC samples indicates good signal stability of the mass spectrum across different time points.

### 2.7. Statistical Analysis

Statistical studies were carried out in order to determine the significance of the variations in lipid content between the three different types of pecans. A one-way analysis of variance (ANOVA) with SPSS 22.0 software was utilized to explore the significant differences in lipid major and subclasses among the three types of pecans. The lipid profiles of various pecans were distinguished using orthogonal partial least squares discriminant analysis (OPLS-DA). The variable importance projection (VIP) values and absolute fold change (FC) values derived from the OPLS-DA model were used to evaluate metabolite differences across groups, and these criteria were used to choose biomarkers for further screening. Furthermore, data were displayed and analyzed using R3.5.1 software.

## 3. Results

### 3.1. Lipid Profiles of Pecan

**Figure 1:**
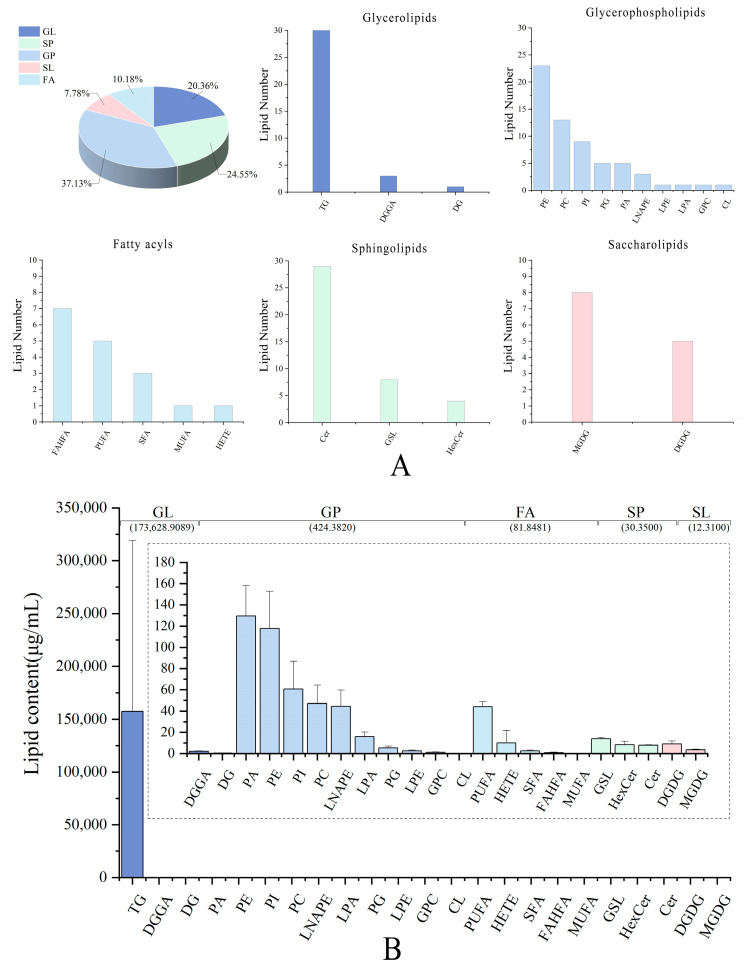
Lipid profile and contents of pecan. (**A**) Percentage and number of individual lipids in pecan from different categories and subclasses. (**B**) The content of individual lipids in pecan from different categories and subclasses. TG, triglyceride; DGGA, diacylglycerylglucuronide; DG, diacylglycerol; PA, phosphatidic acid; PE, glycerophospholipid; PI, phosophatidylinositol; PC, choline glycerophsopholipid; LNAPE, N-acyl-lysophospahtidylethanolamine; LPA, lysophosphatidic acid; PG, phosphatidylglycerol; LPE, lyso-phosphatidylethanolamine; GPC, choline glycerophosphate; CL, cardiolipin; PUFA, polyunsaturated fatty acid; HETE, hydroperoxyeicosatetraenoic acid; SFA, saturated fatty acids; FAHFA, fatty acid ester of hydroxy fatty acid; MUFA, monounsaturated fatty acid; GSL, glycol-sphingolipid; HexCer, hexosylceramide; Cer, ceramide; DGDG, digalactosyldiacylglycerol; MGDG, monogalactosyldiacylglycerol.

Pecan samples contained a total of 167 lipids, with 139 were in the negative ion mode and 28 in the positive ion mode (Figure S2, Table S3). Lipids were divided into five categories [glycerophospholipids (GP), glycerolipids (GL), saccharolipids (SL), sphingolipids (SP), and fatty acyls (FA)] and 23 subclasses. The quantity of lipid molecules in each subclass varied substantially, as shown in Figure 1A. Notably, GP included the greatest lipid molecules, accounting for up to 37.13% of the total amount. There were 10 different types of GPs discovered, with PE being the most prevalent (with 23 different types) and PCs coming in second (with 13 different types) (Figure S3). In addition, SP is in second place with 24.55% of the total amount. There were three sorts of SPs, including 29 ceramides (Cers), 8 glycosphingolipids (GSLs), and 4 hexosylceramide (HexCers). The remaining lipid class accounted for less than 10% of the total, with eight monogalactosyldiacylglycerols (MGDGs) and five digalactosyldiacylglycerols (DGDGs) species.

Previous research has discovered five classes of lipids in pecan [1,10], with GP being the most prevalent major class. In addition, LNAPE, which were partially hydrolyzed products of N-acyl-phospahtidylethanolamine (NAPE), were identified in pecan kernels for the first time. Despite belonging to the phosphatidylethanolamines (PEs) family, the amine moieties of these lipids are esterified with fatty acids [20]. NAPEs are endogenous signaling molecules that are precursors of bioactive N-acylethanolamines (NAE). The incorporation of NAPE-producing bacteria into the gut slows the development of high-fat diet-induced obesity in wild-type mice [21] and improves cardio metabolic disease indicators in low-density lipoprotein null mice [22]. Despite their numerous benefits, NAPEs in food are still poorly understood.

The lipid subclass proportion was determined by summing the quantities of various lipids within each respective subclass. The highest percentage of GPs was found in pecan samples, followed by SPs and GLs, which contributed 25% and 20%, respectively. The one remaining lipid class accounted for less than 10% of the total, with five DGDGs and eight MGDGs species. The identification of triacylglycerols, diacylglycerols, and GPC was predominantly observed in positive mode; negative mode primarily revealed the identification of PE, PI, PG, PA, ceramides, saccharolipids, and fatty acyls.

GPs are the most characterized lipid species, but they are significantly less common than GLs (Figure 1B). The most prudent lipid classes in pecan were GLs. Notably, TGs accounted for 99% of the lipids in pecan kernels, while DGGAs and DGs accounted for 0.1%. The highest in GL are TGs, which are about a thousand times higher than the rest of the subclasses. As the predominant constituent in sebaceous lipids, TGs can exert a substantial influence on the well-being of the skin [23]. Because their carbon chains form a permeable barrier in the epidermis, TGs have physicochemical properties such as barrier function, sunburn, and UV protection [23]. 

### 3.2. The Lipid Compositions of Pecan in Response to Biotic Stress

**Figure 2:**
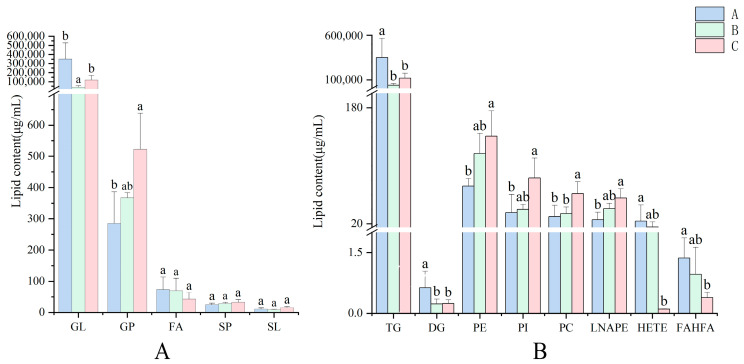
The differences of three types of pecans. (**A**) The difference comparison in lipid concentration among the lipid classes from the three types of pecans. (**B**) The difference comparison in lipid concentration among the lipid subclasses from the three types of pecans. A, Type A; B, TypeB; C, Type C; GL, glycerolipids; GP, glycerophospholipids; FA, fatty acyls; SP, sphingolipids; SL, saccharolipids; TG, triglyceride; DG, diacylglycerol; PE, glycerophospholipid; PI, phosophatidylinositol; PC, choline glycerophsopholipid; LNAPE, N-acyl-lysophospahtidylethanolamine; HETE, hydroperoxyeicosatetraenoic acid; FAHFA, fatty acid ester of hydroxy fatty acid. (a), (ab), (b) means within each column with different letters are significantly (*p* < 0.05) different.

The histogram (Figure 2A) depicts the quantitative variations in lipid categories among the three types of pecans. Non-infected fruit (Type A) clearly beats pecan generated from both types of contaminated fruit (Type B and Type C) in the lipid categories GL and FA. Type C was abundant in GP, SP, and SL, with concentrations of 522.64 μg/mL, 33.14 μg/mL, and 15.23 μg/mL, respectively. In addition, the GP concentration was significantly greater (*p* < 0.05) than the Type B value of 367.87 μg/mL. The majority of lipid subclasses, including TG, PC, PE, PI, and LNAPE, showed significant (*p* < 0.05) alterations. Furthermore, Type A had concentrations of TG, HETE, and FAHFA of 351,706.67, 23.70 and 1.37 μg/mL, respectively, which were 66.21%, 99.58%, and 71.53% greater than Type C. The PI, LNAPE, PC, PE, and PG concentrations of Type C were 2.32, 2.18, 2.07, 1.95, and 1.91 times higher than that of Type A. The PI and PC contents in Type C were higher than those in Type B. Despite the fact that the majority of differences between infested and non-infested fruit were discovered, two lipid subclasses (PI and PC) exhibited significant (*p* < 0.05) differences between non-infested fruits. Individual lipid variation is most common in the LNAPE, FAHFA, and PE categories. 

To evaluate changes in pecan lipids under biotic stress, we measured the content of lipids in each subclass after accumulation. Significant (*p* < 0.05) differences in various lipid subclasses were found between non-infected and infected pecan samples under biotic stress. The overall lipid content was reduced by two-fold. The content of HETE, FAHFA, and TG was reduced by 230.25, 3.54, and 2.96 times, respectively.

### 3.3. The Lipid Chain Length and Unsaturation of Pecan in Response to Biotic Stress

**Figure 3:**
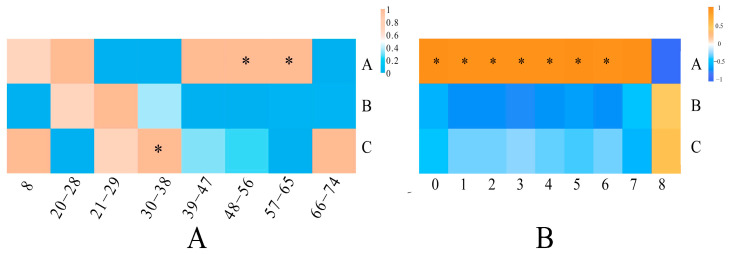
The differences in lipid chain length and unsaturation content of three types of pecans. (**A**) Lipid chain length. (**B**) Lipid unsaturation. A, Type A; B, TypeB; C, Type C. Note: *: there is a significant (*p* < 0.05) difference in the same column and it is the location where the maximum value occurs.

The length of a lipid chain is determined by the total of the carbon atoms in its fatty acid chains. Plant stress resistance is found to be highly related to chain length [24]. This study’s 167 lipids are divided into 8 classes. With average contents of 169,927.20, 421.63, and 394.04 μg/mL, the top three chain length classes are 48–56, 57–65, and 30–38. The highest variation (95.10%) in lipid content was found between 48–56 chain lengths among the three types of pecans. Infested pecans (Type C) showed the highest contents in the majority of the chain length groupings, especially those with fewer than 48 chains (Figure 3A). The chain length of Type C was 36 of 317.82 μg/mL, which was 1.92 and 1.51 times higher than non-infested pecans from non-infested fruits (Type A) and infested pecans from non-infested fruits (Type B). Moreover, Type C was found to be substantially greater (*p* < 0.05) in the chain lengths of 30–38 than in non-infested fruit, with an average level of 528.86 μg/mL. The average contents of non-infested pecans at chain length ranges of 48–56 and 57–65 were 350,955.94 μg/mL and 750.36 μg/mL, which were significantly (*p* < 0.05) greater than the infested fruit. However, there was no significant (*p* > 0.05) difference between the two samples of infested fruits for any chain length group. In addition, the lipid composition discrepancies between the three types of pecans were subsequently evaluated at each degree of unsaturation (Figure 3B). Type A exhibited significantly (*p* < 0.05) higher levels of chain unsaturation degrees 1–6 compared to the other types. However, at chain unsaturation degree 8, Type C demonstrated a 2.49-fold increase in comparison to the non-infested branch (Type A).

### 3.4. Multivariate Statistical Analysis

**Figure 4:**
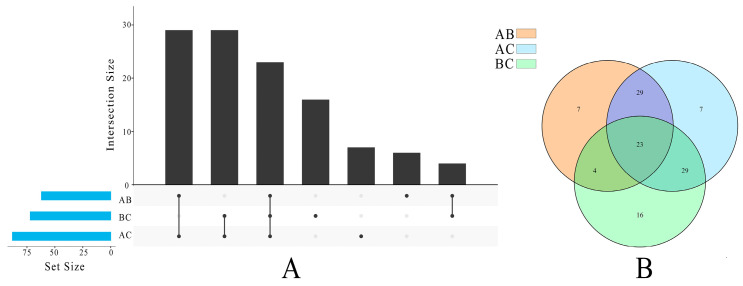
Diagram of lipidomics distinguishes any two types of pecans: (**A**) histogram; (**B**) Venn diagram.

We employed a multivariate statistical analysis in this work to investigate the variations in the lipid profiles of different pecan types. By utilizing robust mathematical models, multivariate statistical analysis simplifies the examination of intricate multivariate data by condensing the characteristics of metabolic profiles. The OPLS-DA model is a supervised method for finding biomarkers that employs a log transformation of the data; the OPLS-DA results diagram clearly reveals that the principal components divide the nut’s lipids into two parts: Type A samples on the left and Types B and C samples on the right (Figure S4). It was revealed that the lipid profiles of infected and non-infected pecans were significantly different. As shown in Figure 4A, a total of 114 individual lipid variables were found to have significant contributions (VIP > 1). Among these, 63 lipids could be distinguished between Type A vs. Type B, 72 lipids between Type B vs. Type C, and 88 lipids between Type A vs. Type C. Furthermore, there were 23 lipids that exhibited distinguishable characteristics between any two types, indicating their potential as important differentially metabolized lipids following insect stress. Out of these 23 lipids, there were 15 TGs (TG 16:0_18:2_18:2, TG 18:1_18:1_18:1, and TG 18:2_18:3_18:3), 5 PEs (PE 16:0_18:3, PE 22:0_18:3, and PE 18:2_18:2), 1 HexCer [HexCer 18:1;3O/26:0;(2OH)], 1 LNAPE (LNAPE 18:2/N-18:2), and 1 PUFA [11(Z),14(Z)-Eicosadienoic Acid] (Figure S5). Individual lipids could also be utilized to distinguish three types of pecans.

## 4. Discussion

Using lipidomic analysis, we investigated the changes in lipids in pecans in response to biotic stress in our current study. There were 167 lipids discovered and quantified in total, with five major categories and 23 subcategories. The lipid composition of pecans was as follows: GL > GP > FA > SP > SL. TG were the most common types of lipid components in GL, while GP were the most diverse, which is in keeping with the findings of a previous study on the distribution of pecan storage lipid species [1,10,14]. Throughout the storage phase, temperature and other stresses raised the intensities of most glycerophospholipid subclasses, including PC, PE, PI, PG, and cardiolipin [10]. However, under biotic stress, the overall lipid content decreased, particularly the levels of TG, DG, HETE, and FAHFA lipids (*p* < 0.05). As a result, the effects of biotic stress on lipid formation may differ significantly from those of other abiotic stresses. Several studies have linked lipid chain length to abiotic stressors in plants [24,25]. The current study also found a significant increase (*p* < 0.05) in PI classes with chain lengths ranging from 30 to 38, such as PI (18:0/18:2), PI (18:1/18:2) and PI (16:0/18:3).

When Type A was compared to Type C, the content of chain lengths longer than 48 lipids (primarily TG) was reduced by 66.07%. The findings showed that insect stress decreased the amount of long-chain lipids in pecans, whereas we previously discovered that insect stress significantly increased the content of long-chain lipids in camellia seeds [15]. Insect stimulation has been shown to increase the accumulation of long-chain lipids in *Arabidopsis thaliana* [26] and *Jatropha curcas* [27] research. The findings show that various plant TG components, including pecan, respond to insect pest stress; however, the impact differs by plant species.

In addition, differences in TG content and structure may play a role in the nutritional and physical characteristics of non-infected and infected pecans. The OPLS-DA results support this observation. A total of 114 VIPs (including 26 TGs) are used to distinguish the three types. The Venn diagram (Figure 4B) depicts the number of subjects with effective discrimination (VIP > 1) in pecan mixtures. Insect stress significantly decreased TG in Type C (*p* < 0.05), and the top three lipid molecules in TG content (18:0_18:1_18:2) had an average VIP value of 1.53. During storage, similar findings were made in hazelnut oil [28] and walnut [29]. TG lipolysis is thought to be a stress response mechanism in woody oilseed crops. However, while TG was significantly reduced during pecan storage, it was discovered that the GL class contributed the most to differentiating lipid changes in pecan kernels throughout storage [10]. 

It is noteworthy that 52 lipids (VIP > 1) significantly contributed to the differentiation of Type C and A, as well as Type C and B. PC (34:2) had the most variation (64.41%), but it also contributed the most to both judgments, with an average VIP value of 1.53. Moreover, because Type B (from infested fruit) and Type A (from perfectly non-infested pecan) are both derived from non-infested pecans, they can be distinguished from each other. The key distinction between them was PE (40:3). PC and PE are both GP phospholipids that are plentiful in pecans. GPs, like phosphatidic acid (PA), phosphatidylinositol (PI), and lysophospholipids (LPs), are flexible signal molecules involved in a variety of stress events such as drought, salt, and heat shock [30]. The phospholipid content of plants increases by about 10% when exposed to cold, non-freezing temperatures [31]. Similar changes were observed in the current study under biotic stress. The GP content in the infested Type C group increased by 83.35% when compared to Type A without infection.

The insect stress resulted in elevated components of all subclasses of GP in pecan. GP is a lipid involved in cell membrane defense. PA, a subclass of GP, is also known to play an important role in transmembrane lipid transport, as well as a precursor of PI, PC, and PE [32]. Apparently, insect stress may have prompted most of the GP subclasses in pecan to perform cytoprotective effects in response to external stimuli, which is also a sign of plant defense [33]. SP controls numerous biological processes, including cell proliferation, differentiation, and death [34]. The SP pathway relies on Cer as its main metabolic hub, which can undergo conversions to HexCer, phSM, and SPH, and different physiological and pathological processes and disorders have been associated with variations in HexCer levels [28]. These findings could explain why HexCer levels rise during biotic stress. Previous studies on GL have shown its potential to enhance cardiovascular health and reduce the risk of heart disease [35]. However, GL levels in Type C were reduced by more than 80% compared to Type A fruits. This implies that insect stress caused greater damage to the function of GL in pecans. In addition, FA in fruit seed and oil has antioxidant and anti-inflammatory effects [35]. Notably, FAHFA, a subclass of FA, has been shown to have positive effects on diseases such as diabetes and inflammation when consumed in the diet [36]. In this study, it was found that insect stress also reduced these nutrients in pecans. This suggests that insect infestation may lead to impairment of the corresponding functions of pecans.

**Figure 5:**
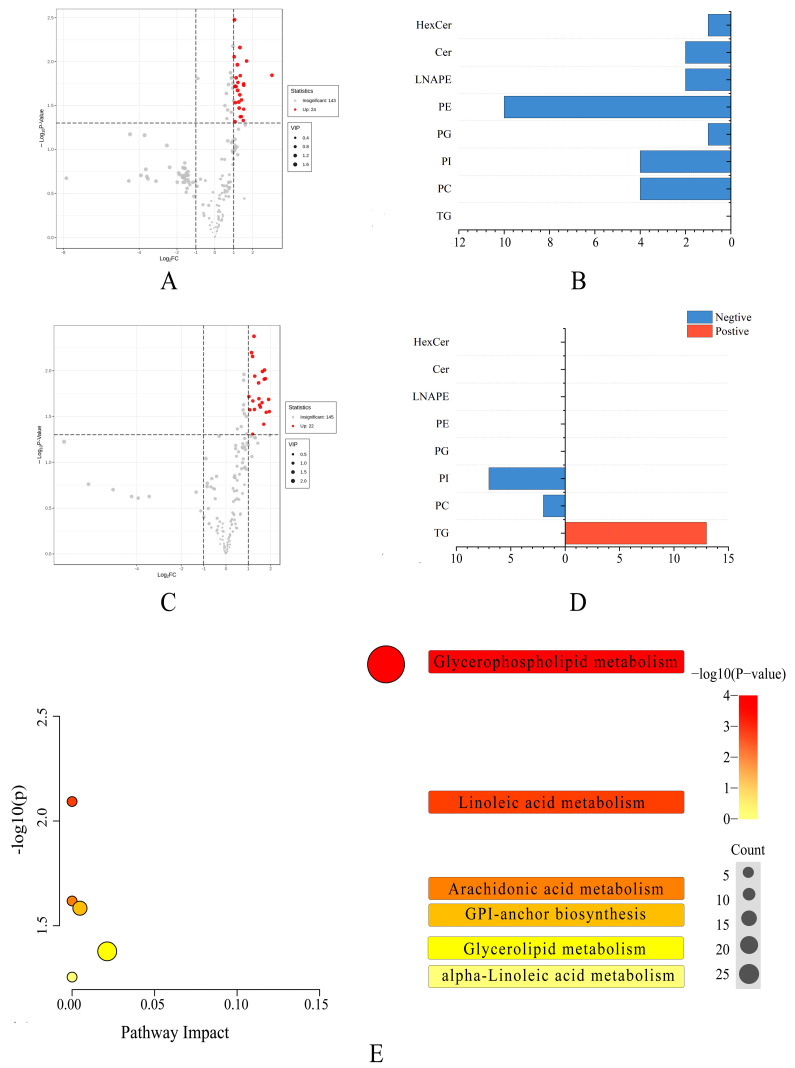
Differential metabolic analysis in response to insect damage. (**A**) Volcano diagram of the differential metabolites of Type A and Type C. (**B**) Numbers of the differential metabolites of Type A and Type C under positive and negative modes. (**C**) Volcano diagram of the differential metabolites of Type B and Type C. (**D**) Numbers of the differential metabolites of Type B and Type C under positive and negative modes. (**E**) Pathway impact diagram. The bubble size represents the number of different metabolites involved in this pathway, and the bubble color represents the *p*-value of this metabolic pathway.

Many previous studies have screened some metabolites as biomarkers for various abiotic stresses, using more stringent screening parameters such as fold change ≥ 2 or fold change ≤ 0.5, *p*-value < 0.05, and VIP ≥ 1 [37,38]. In this study, we used these stricter criteria to identify potential biomarkers. We discovered only three distinct metabolites between Type A and Type B, indicating that there are no significant differences in metabolite profiles between the two pecan varieties. Moreover, 44 different metabolites have been identified in the other two comparison groups, including 24 in the Type A vs. Type C group and 22 in the Type B vs. Type C group. The top three molecules with high VIP values in the Type A vs. Type C group are PE 22:0_18:3 (1.55), PE 22:0_18:2 (1.52), and Cer 18:0;3O/26:0 (1.46); and in the Type B vs. Type C group, they are PI 18:0_18:2 (1.69), PI 18:1_18:2 (1.63), and PI 18:0_18:3 (1.62). The differential metabolites found in the Type A vs. Type C group were all detected in the negative ion mode and belonged primarily to the GP class, whereas the differential metabolites found in the Type B vs. Type C group were all detected in the positive ion mode and belonged primarily to the TG subclass (Figure 5B,D). Previous studies have shown that TG is a lipid species that changes significantly during hickory (belong to *Carya* Nutt.) development, suggesting that TG is a sensitive lipid species in response to environmental changes or the plant’s own physiological properties [14]. In our study, we also revealed that TG is sensitive in response to insect stress in pecans. Notably, two PI [PI (34:3), PI (36:4)] differential metabolites separated from the two groups (Type A vs. Type C and Type B vs. Type C) in our investigation.

Using these indicators, we investigated the mechanisms of pecan metabolic disruption caused by insect stress. Glycerophospholipid metabolism, linoleic acid metabolism, arachidonic acid metabolism, glycosylphosphatidylinositol (GPI), anchor biosynthesis, and glycerolipid metabolism were five of the most significant (*p* < 0.05) pathways discovered (Figure 5E). Glycerophospholipid metabolism, such as hazelnut during storage and camellia seeds under biotic stress, had the greatest impact [15,39]. Pecan has 62 different kinds of GPs. Despite the low GP content, the quantity of GPs varies significantly in response to biotic stress, especially for PE. PE is required for the synthesis of glycosylphosphatidylinositolanchored protein (GPI-AP), which is necessary for cell viability [40]. Furthermore, glycolipid metabolism is critical under biotic stress. Among the various lipids, there are 13 kinds of GLs, and these neutral lipids participate in a variety of key metabolic processes. The DG generated from the breakdown of TG acts as a precursor for the synthesis of PC and PE through the Kennedy pathway, while also serving as a secondary messenger in signal transduction by binding to protein kinases [41]. A variety of significant disorders have been connected to the accumulation and breakdown of neutral lipids. Overall, the current study’s findings demonstrated a change in the lipid profile of pecans under biotic stress, offering fresh insight into the influence of biotic stress on oily nuts. 

## 5. Conclusions

A lipidomic approach was used to assess changes in the lipid profile of pecans from infested and uninfested fruits in our current study. Pecan contained 167 individual lipids from five lipid categories (GP, GL, SL, SP, and FA) and 23 subclasses. The study’s findings indicate that biotic stress has a significant effect on GP and GL, significantly reducing TG lipids with chain lengths greater than 48. *Conogethes punctiferalis* infestation led to an increase in GP content, especially PE, PA, and PI, and the changes in these subclasses may be the result of chemical defense by pecan. Insect stress reduces the GL and FA content of pecans, which adversely affects their food nutrition. Based on bioinformatics analysis, 44 lipid monomers were discovered to respond to insect pests and can be used as potential biomarkers to differentiate infected and non-infected pecans. PI (18:0_18:2), PI (18:1_18:2), PI (18:0_18:3) TG (18:0_18:1_18:2), TG (18:1_18:2_18:3), TG (16:0_16:0_18:1), PE (22:0_18:3), TG (16:0_18:1_18:3), PE (22:0_18:2), and TG (18:1_18:1_18:1) have the highest VIP (>1.5). Six metabolic disruption pathways of peach stem borer by pecans were identified, with glycerophospholipid metabolism being the most important. We comprehensively analyzed pecan lipidomics under insect stress to better understand the complicated relationships between insects and pecans. This study emphasizes the significance of lipid-related signaling and defense pathways in pecan tree adaptation to biotic challenges, giving insights into the nut’s resistance and future pest control and pecan crop enhancement opportunities.

## Data Availability

The original contributions presented in the study are included in the article/Supplementary Material, further inquiries can be directed to the corresponding authors.

## Supplementary Materials

The following supporting information can be downloaded at https://www.mdpi.com/article/10.3390/foods13070974/s1: Figure S1: The different types of pecans; Figure S2: Numbers of lipids identified under positive and negative modes; Figure S3: The content of individual lipids in pecan oils from different subclasses; Figure S4: OPLS-DA scores plot. (A) Type A and Type B. (B) Type A and Type C. (C) Type B and Type C; Figure S5; VIP scores of individual lipids in OPLS-DA; Table S1: Details of internal standards; Table S2: Instrument parameters and programmer settings; Table S3: Details of 167 lipids.
