# Supplementary material for: Evaluation of Lipid Quality in Fruit: Utilizing Lipidomic Approaches for Assessing the Impact of Biotic Stress on Pecans (Carya illinoinensis)
Source: Foods. 2024 Mar 22;13(7):974. doi: 10.3390/foods13070974 (PMC11011906; doi:10.3390/foods13070974)
Supplement: Supplementary file 1 [file foods-13-00974-s001.zip › foods-2904685-supplementary.pdf]

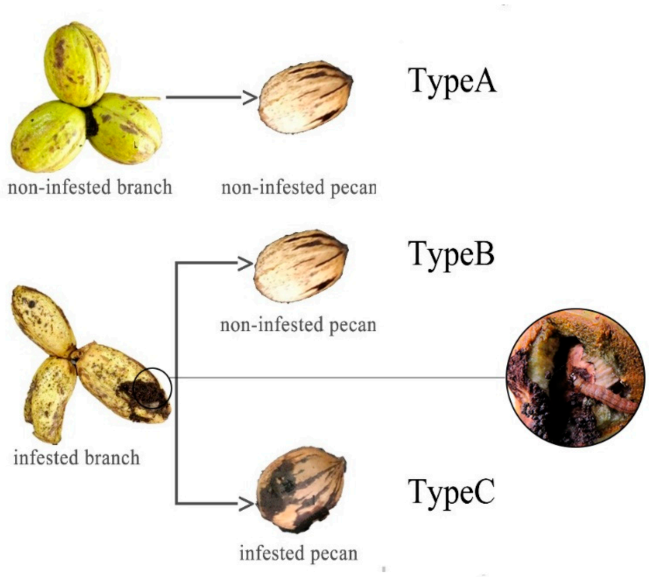

Figure S1. The different types of pecans.

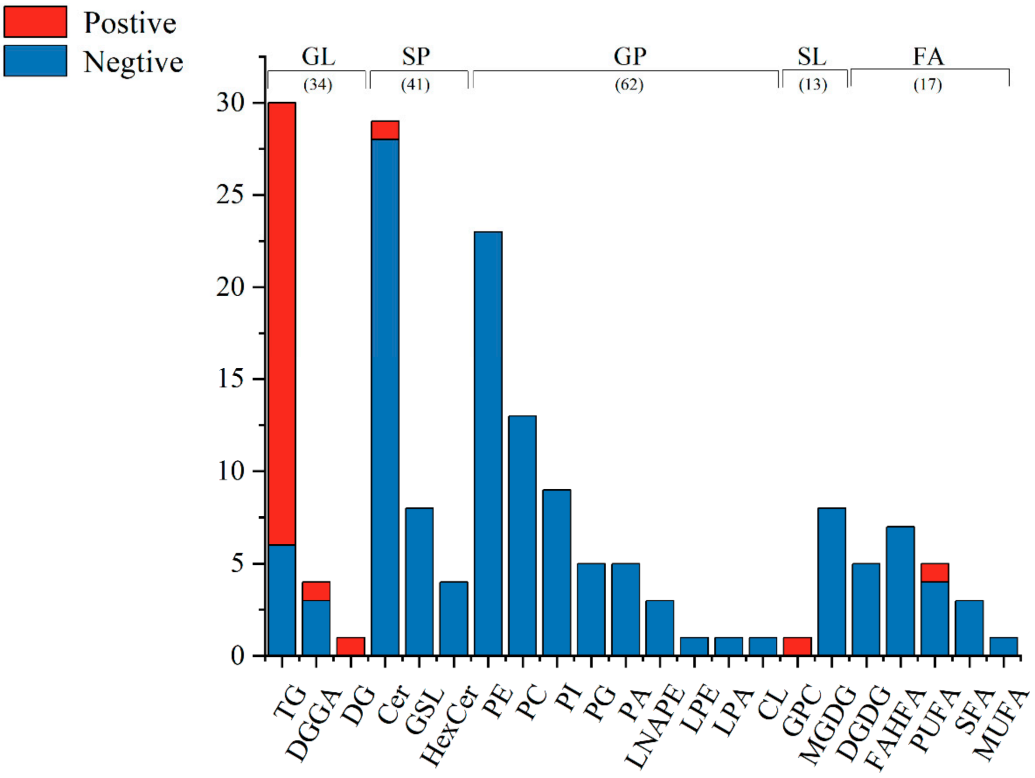

Figure S2. Numbers of lipids identified under positive and negative modes.

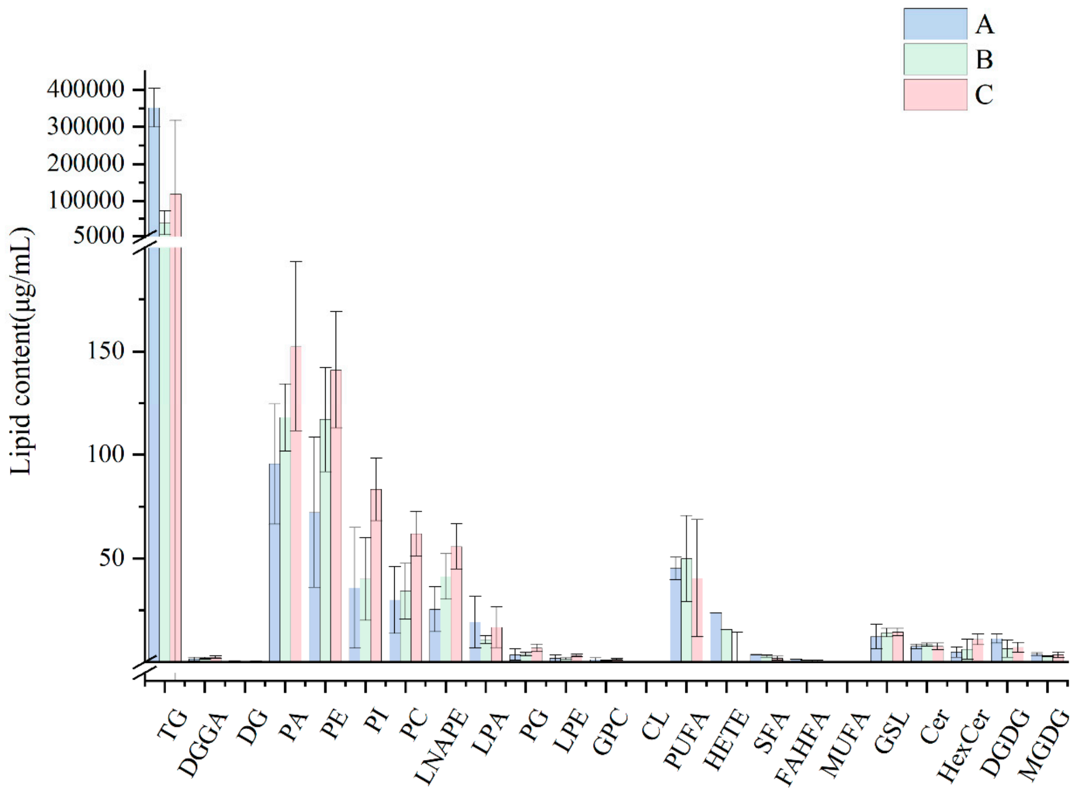

Figure S3. The content of individual lipids in pecan oils from different subclasses.

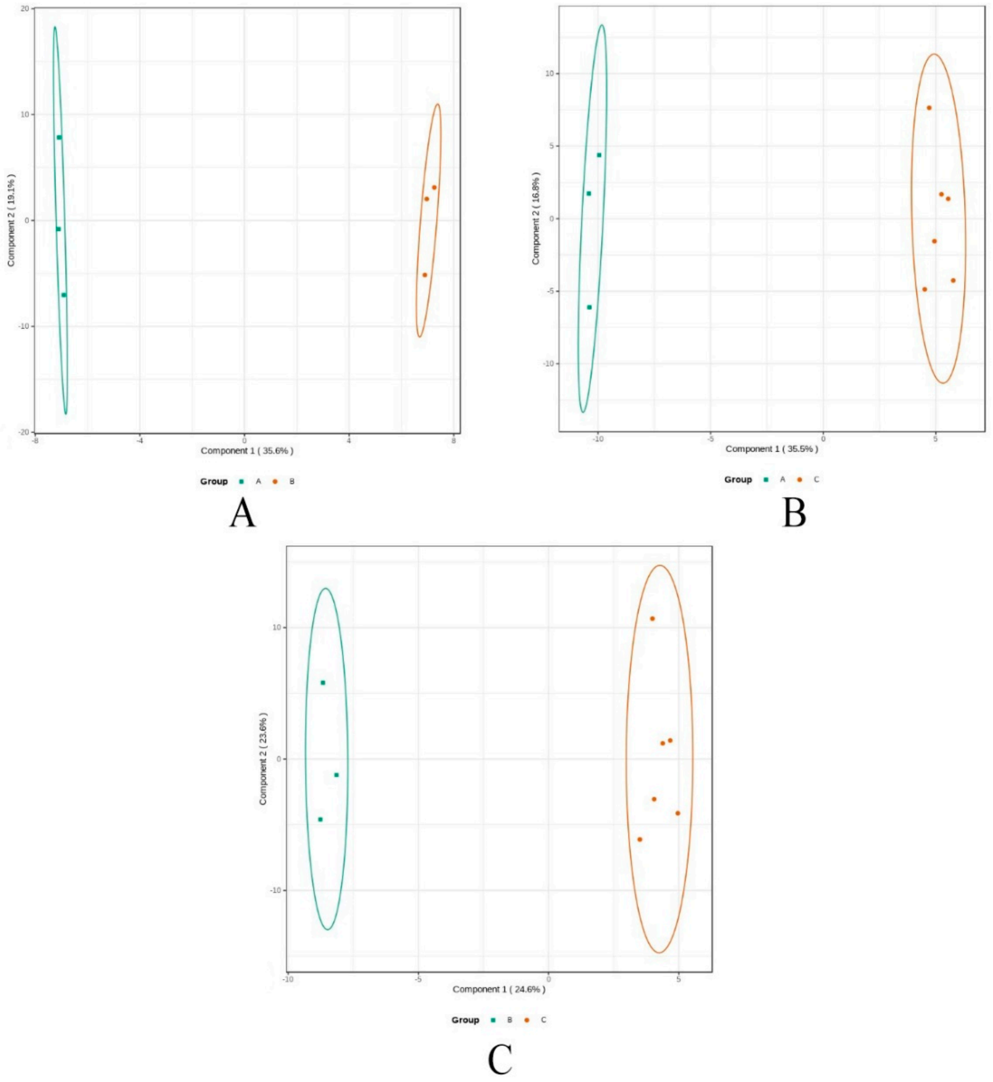

Figure S4. OPLS-DA scores plot. (A) Type A and Type B. (B) Type A and Type C. (C) Type B and Type C.

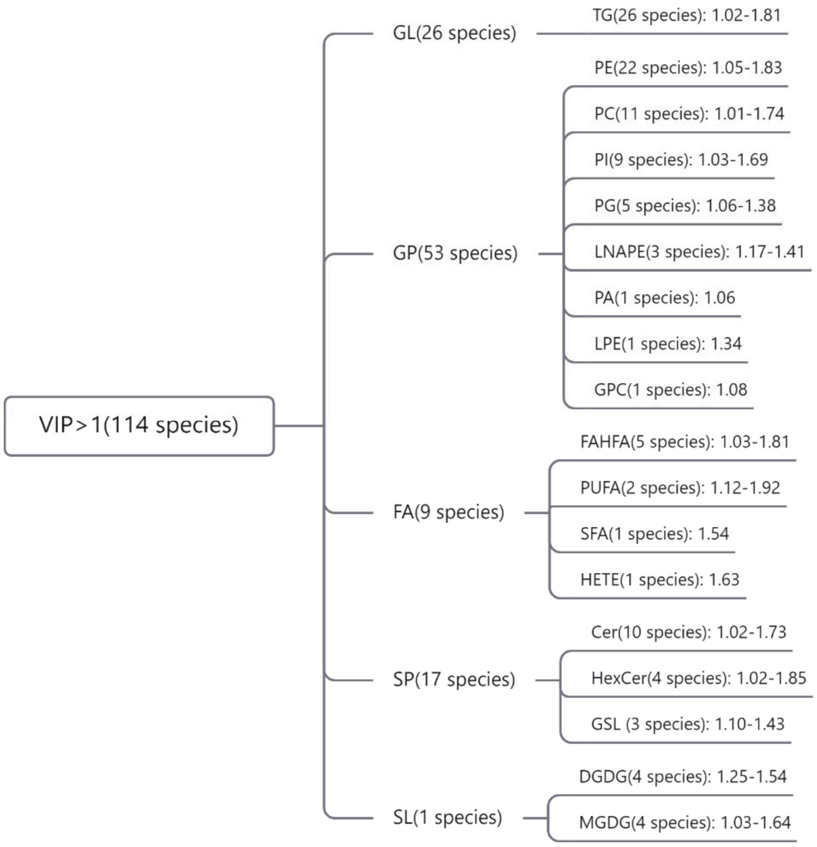

Figure S5. VIP scores of individual lipids in OPLS-DA.

GL, glycerolipids; GP, glycerophospholipids; FA, fatty acyls; SP, sphingolipids; SL, saccharolipids.

Table S1. Details of internal standards.

Internal standards	Producers
PC 15:0-18:1-d7	Avanti Polar Lipids (Alabaster, Alabama, USA)
PG 15:0-18:1-d7	Avanti Polar Lipids (Alabaster, Alabama, USA)
PE 15:0- 18:1-d7	Avanti Polar Lipids (Alabaster, Alabama, USA)
LPC 18:1-d7	Avanti Polar Lipids (Alabaster, Alabama, USA)
LPE 18:1-d7	Avanti Polar Lipids (Alabaster, Alabama, USA)
SM d18:1/18:1-d9	Avanti Polar Lipids (Alabaster, Alabama, USA)
TAG 15:0-18:1-d7-15:0	Avanti Polar Lipids (Alabaster, Alabama, USA)

Table S2. Instrument parameters and programmer settings.

Instrument	Programmer and parameter settings
Thermo Fisher Ultimate 3000 UHPLC system	Waters CSH C18 column: 2.1mm × 100 mm, 1.7 μm. The ultra-pure water (A) and isopropanol/acetonitrile (9:1, v/v) (B), each containing 10 mM ammonium formate and 0.1% formic acid, were utilized as mobile phases of positive ionization mode, while the same mobile phases except for formic acid were applied as those of negative ionization mode. A linear elution program was performed to separate lipids as the following: 40% B at 0 min; 45% B at 2 min; 55% B at 4 min and held until 10 min; 90% B at 14 min; 95% B at 15 min and held until 18 min; 40% B at 18.1 min and held until 20 min. The flow rate was selected to be 0.3 mL/min and the injection volume was set to 2 μL.
ThermoFisher Q Exactive Hybrid Quadrupole-Orbitrap Mass Spectrometry Mass Spectrometer	The eluted lipids were analysed by heated electrospray ionization in both positive and negative ionisation modes. The spray voltage was 3.5 kV, and the temperatures of capillary and auxiliary gas were selected to be 350 °C. The flow rates of sheath gas were 40 (Arb) and auxiliary gas were10 (Arb). The S-Lens RF Level was 50 (Arb). Full scan analysis was performed at a high-resolution (70,000 FWHM at m/z = 200) within m/z 130 - 1950, with an AGC Target setting of 1×10^6. Each scan was set with a mass resolution of 17,500 and an AGC target of 5 × 10^5 for the acquisition of fragment ion information for the 10 former source ions. Data-dependent acquisition were selected with HCD energy set at NCE 20, 30, and 40.

Table S3. Details of 167 lipids.

Name	ClassI	ClassII	m/z	Adduct
Linoleic acid	FA	PUFA	279.2331	[M-H]-
15(S)-HpETE	FA	HETE	335.2232	[M-H]-
Docosanoic acid	FA	SFA	339.3268	[M-H]-
8Z,11Z,14Z-Eicosatrienoic acid	FA	PUFA	305.2487	[M-H]-
NAGly 22:0/18:0	FA	PUFA	680.6191	[M+NH4]+
Arachidonic acid	FA	PUFA	303.2331	[M-H]-
11(Z),14(Z)-Eicosadienoic Acid	FA	PUFA	307.2644	[M-H]-
Myristic Acid	FA	SFA	227.2017	[M-H]-
FAHFA 14:0/14:0	FA	FAHFA	453.3954	[M+FA-H]-
Lignoceric acid	FA	SFA	367.3582	[M-H]-
11(E)-Eicosenoic Acid	FA	MUFA	309.2800	[M-H]-
FAHFA 18:2/18:1	FA	FAHFA	559.4735	[M-H]-
FAHFA 18:1/22:0	FA	FAHFA	619.5678	[M-H]-
FAHFA 18:2/22:0	FA	FAHFA	617.5521	[M+FA-H]-
FAHFA 16:0/22:0	FA	FAHFA	593.5522	[M+FA-H]-
FAHFA 16:0/18:1	FA	FAHFA	535.4737	[M-H]-
FAHFA 18:1/18:2	FA	FAHFA	559.4736	[M-H]-
TG 18:0_18:1_18:2	GL	TG	902.8176	[M+NH4]+

TG 18:1_18:1_18:2	GL	TG	900.8011	[M+NH4]+
TG 16:0_18:1_18:1	GL	TG	876.8014	[M+NH4]+
TG 16:0_16:0_18:1	GL	TG	850.7867	[M+NH4]+
TG 16:3_20:0_20:1	GL	TG	928.8326	[M+NH4]+
TG 18:1_18:2_20:1	GL	TG	928.8325	[M+NH4]+
TG 18:2_18:2_20:1	GL	TG	926.8168	[M+NH4]+
TG 18:2_18:3_20:0	GL	TG	926.8167	[M+NH4]+
TG 16:0_18:1_18:3	GL	TG	877.7259	[M+NH4]+
TG 16:0_16:0_18:2	GL	TG	848.7711	[M+NH4]+
TG 18:1_18:1_24:0	GL	TG	988.9267	[M+NH4]+
TG 17:1_18:2_18:2	GL	TG	889.7266	[M+NH4]+
TG 14:1_18:1_18:1	GL	TG	846.7549	[M+NH4]+
TG 18:1_18:2_20:5	GL	TG	920.7707	[M+NH4]+
DGGA 18:2_18:2	GL	DGGA	791.5325	[M+FA-H]-
TG 16:0_16:0_16:0	GL	TG	824.7707	[M+NH4]+
TG 18:2_18:3_18:3	GL	TG	829.7281	[M+NH4]+
DG 20:0_18:2	GL	DG	634.5780	[M+NH4]+
TG 9:0_18:2_18:2	GL	TG	750.6608	[M+NH4]+
TG 18:1_18:2_18:3	GL	TG	901.7248	[M+Na]+
TG 16:0_18:2_18:2	GL	TG	872.7711	[M+Na]+

TG 18:1_18:2_18:2	GL	TG	903.7405	[M+Na]+
TG 18:1_18:1_18:1	GL	TG	907.7720	[M+Na]+
TG 16:0_18:1_18:2	GL	TG	874.7864	[M+Na]+
TG 18:2_18:2_18:3	GL	TG	894.7550	[M+Na]+
DGGA 18:2_18:3	GL	DGGA	789.5171	[M+FA-H]-
ADGGA 18:2_18:2_18:2	GL	TG	1053.7618	[M-H]-
DGGA 18:1_18:1	GL	DGGA	795.5639	[M+FA-H]-
ADGGA 18:2_16:0_18:2	GL	TG	1029.7620	[M-H]-
ADGGA 18:1_16:0_18:2	GL	TG	1031.7772	[M-H]-
TG 14:0_16:0_18:2	GL	TG	820.7399	[M+Na]+
ADGGA 18:2_18:2_18:3	GL	TG	1051.7458	[M-H]-
ADGGA 16:0_18:2_18:3	GL	TG	1027.7460	[M-H]-
ADGGA 18:1_16:0_16:0	GL	TG	1007.7789	[M+FA-H]-
PA 16:0_18:2	GP	PA	671.4668	[M-H]-
PA 18:1_18:2	GP	PA	697.4821	[M-H]-
PE 16:0_18:2	GP	PE	714.5087	[M-H]-
LNAPE 18:2/N-18:2	GP	LNAPE	738.5088	[M-H]-
PE 18:2_18:2	GP	PE	738.5089	[M-H]-
PI 16:0_18:2	GP	PI	833.5196	[M-H]-
PC 18:2_18:2	GP	PC	826.5616	[M+FA-H]-

PE 18:1_18:2	GP	PE	740.5245	[M+FA-H]-
LPA 18:2	GP	LPA	433.2364	[M-H]-
PC 18:1_18:2	GP	PC	828.5771	[M+FA-H]-
PI 18:0_18:2	GP	PI	861.5507	[M-H]-
PA 18:2_18:3	GP	PA	693.4509	[M-H]-
PI 18:1_18:3	GP	PI	857.5194	[M-H]-
PI 18:1_18:2	GP	PI	859.5350	[M-H]-
PI 16:0_18:1	GP	PI	835.5353	[M-H]-
PG 16:0_18:1	GP	PG	747.5193	[M-H]-
LPE 18:2	GP	LPE	476.2786	[M-H]-
PE 18:2_18:3	GP	PE	736.4933	[M-H]-
PI 16:0_18:3	GP	PI	831.5040	[M-H]-
PA 18:2_18:2	GP	PA	695.4665	[M-H]-
PE 16:0_18:3	GP	PE	712.4932	[M+FA-H]-
PI 18:0_18:1	GP	PI	863.5664	[M+FA-H]-
PC 18:2_18:3	GP	PC	824.5461	[M+FA-H]-
PE 16:0_18:1	GP	PE	716.5245	[M+FA-H]-
PE 22:0_18:2	GP	PE	798.6030	[M+FA-H]-
PG 16:0_16:0	GP	PG	721.5035	[M+FA-H]-
PE 18:0_18:2	GP	PE	742.5404	[M+FA-H]-

PEtOH 18:2_18:2	GP	PE	723.4982	[M-H]-
PC 16:0_18:1	GP	PC	804.5771	[M+FA-H]-
PC 16:0_18:3	GP	PC	800.5460	[M+FA-H]-
PC 18:0_18:2	GP	PC	830.5930	[M+FA-H]-
PG 18:2_18:2	GP	PG	769.5034	[M+FA-H]-
PI 18:0_18:3	GP	PI	859.5351	[M-H]-
PEtOH 16:0_18:2	GP	PE	699.4981	[M-H]-
PG 18:1_18:2	GP	PG	771.5190	[M-H]-
PG 18:0_18:2	GP	PG	773.5346	[M+FA-H]-
PA 22:0_18:2	GP	PA	755.5605	[M-H]-
PC 16:0_16:0	GP	PC	778.5615	[M+FA-H]-
PC 18:1_18:1	GP	PC	830.5929	[M+FA-H]-
PEtOH 18:1_18:2	GP	PE	725.5139	[M-H]-
LNAPE 18:1/N-18:1	GP	LNAPE	742.5403	[M+FA-H]-
PE 20:0_18:2	GP	PE	770.5715	[M+FA-H]-
PC 18:0_18:1	GP	PC	832.6084	[M+FA-H]-
PE 24:0_18:2	GP	PE	826.6345	[M-H]-
PI 22:0_18:2	GP	PI	917.6137	[M-H]-
PE 18:0_18:1	GP	PE	744.5560	[M-H]-
PE 23:0_18:2	GP	PE	812.6187	[M+FA-H]-

PC 16:0_18:0	GP	PC	806.5930	[M+FA-H]-
PEtOH 18:1_18:1	GP	PE	727.5292	[M+FA-H]-
PE 21:0_18:2	GP	PE	784.5875	[M-H]-
PE 17:0_18:2	GP	PE	728.5247	[M+FA-H]-
CL 18:2_18:2_18:2_18:2	GP	CL	1447.9665	[M+FA-H]-
HBMP 18:1_18:2_16:0	GP	PC	1009.7488	[M+FA-H]-
PE 22:0_18:1	GP	PE	800.6187	[M+FA-H]-
LNAPE 16:0/N-16:0	GP	LNAPE	690.5088	[M+FA-H]-
HBMP 16:0_18:2_16:0	GP	PC	983.7327	[M-H]-
PEtOH 16:0_18:1	GP	PE	701.5136	[M+FA-H]-
PE 22:0_18:3	GP	PE	796.5873	[M+FA-H]-
PC 16:0_18:2	GP	PC	802.5617	[M+FA-H]-
PE 25:0_18:2	GP	PE	840.6497	[M+FA-H]-
Glycerophosphocholine	GP	GPC	258.1111	[M+H]+
PE 22:1_18:2	GP	PE	796.5872	[M+FA-H]-
DGDG 18:2_18:2	SL	DGDG	985.6121	[M+FA-H]-
MGDG 18:2_18:2	SL	MGDG	823.5592	[M+FA-H]-
DGDG 18:2_18:3	SL	DGDG	983.5964	[M+FA-H]-
DGDG 16:0_18:2	SL	DGDG	961.6120	[M+FA-H]-
DGDG 18:1_18:2	SL	DGDG	987.6277	[M+FA-H]-

MGDG O-28:4_18:1	SL	MGDG	947.7204	[M+FA-H]-
MGDG 18:2_18:3	SL	MGDG	821.5449	[M+FA-H]-
MGDG 18:1_18:2	SL	MGDG	825.5748	[M+FA-H]-
MGDG 18:3_18:3	SL	MGDG	819.5290	[M+FA-H]-
MGDG 16:0_18:2	SL	MGDG	799.5589	[M+FA-H]-
MGDG O-26:1_18:2	SL	MGDG	923.7211	[M+FA-H]-
MGDG 18:1_18:1	SL	MGDG	827.5904	[M+FA-H]-
DGDG 16:0_18:1	SL	DGDG	963.6277	[M+FA-H]-
HexCer 22:1;2O/12:1;O	SP	HexCer	758.5432	[M+FA-H]-
ST 29:1;O;Hex;FA 16:0	SP	GSL	859.6678	[M+FA-H]-
ST 28:1;O;Hex;FA 18:1	SP	GSL	885.6835	[M+FA-H]-
ST 29:1;O;Hex;FA 18:0	SP	GSL	887.6993	[M+FA-H]-
Cer 18:0;3O/16:0	SP	Cer	600.5216	[M+FA-H]-
Cer 38:2;2O/2:0	SP	Cer	620.5983	[M+NH4]+
Cer 18:0;3O/24:0	SP	Cer	712.6471	[M+FA-H]-
Cer 18:1;3O/16:0	SP	Cer	598.5063	[M+FA-H]-
ST 29:2;O;Hex;FA 18:2	SP	GSL	881.6523	[M+FA-H]-
Cer 18:1;3O/24:0	SP	Cer	710.6313	[M+FA-H]-
Cer 18:1;3O/22:0	SP	Cer	682.6000	[M+FA-H]-
HexCer 18:1;3O/22:0;(2OH)	SP	HexCer	860.6476	[M+FA-H]-

Cer 18:0;2O/16:0	SP	Cer	584.5266	[M+FA-H]-
HexCer 18:1;3O/24:0;(2OH)	SP	HexCer	888.6794	[M+FA-H]-
ST 27:1;O;Hex;FA 16:0	SP	GSL	845.6523	[M+FA-H]-
Cer 18:2;2O/22:0;(2OH)	SP	Cer	680.5844	[M+FA-H]-
ST 28:2;O;Hex;FA 18:1	SP	GSL	869.6521	[M+FA-H]-
ST 28:2;O;Hex;FA 16:0	SP	GSL	857.6522	[M+FA-H]-
Cer 18:0;3O/25:0	SP	Cer	726.6630	[M+FA-H]-
Cer 18:2;2O/18:2	SP	Cer	604.4955	[M+FA-H]-
Cer 18:1;3O/24:0;(2OH)	SP	Cer	726.6264	[M+FA-H]-
Cer 18:1;3O/25:0	SP	Cer	724.6472	[M+FA-H]-
Cer 18:0;3O/26:0	SP	Cer	740.6786	[M+FA-H]-
Cer 18:0;3O/20:0	SP	Cer	656.5845	[M+FA-H]-
Cer 18:2;2O/25:0;(2OH)	SP	Cer	722.6316	[M+FA-H]-
Cer 18:2;2O/24:0;(2OH)	SP	Cer	662.6094	[M-H]-
Cer 18:2;2O/24:0	SP	Cer	692.6207	[M+FA-H]-
ST 29:1;O;Hex;FA 18:2	SP	GSL	883.6680	[M+FA-H]-
Cer 18:1;3O/21:0	SP	Cer	668.5849	[M+FA-H]-
Cer 18:1;3O/20:0	SP	Cer	654.5691	[M+FA-H]-
Cer 18:2;2O/18:1	SP	Cer	606.5113	[M+FA-H]-
Cer 18:0;3O/18:0	SP	Cer	628.5531	[M+FA-H]-

Cer 18:0;3O/24:0;(2OH)	SP	Cer	728.6422	[M+FA-H]-
Cer 18:0;2O/18:0	SP	Cer	612.5579	[M+FA-H]-
Cer 18:2;2O/23:0	SP	Cer	678.6052	[M+FA-H]-
Cer 21:0;2O/12:0;(3OH)(FA 18:2)	SP	Cer	862.7517	[M+FA-H]-
HexCer 18:1;3O/26:0;(2OH)	SP	HexCer	916.7105	[M+FA-H]-
Cer 22:0;2O/16:3;(3OH)(FA 18:1)	SP	Cer	928.7989	[M+FA-H]-
Cer 18:1;3O/24:1	SP	Cer	708.6158	[M+FA-H]-
Cer 18:1;3O/24:1;(2OH)	SP	Cer	724.6108	[M+FA-H]-
Cer 18:0;3O/24:1	SP	Cer	710.6315	[M+FA-H]-
